# 839. Insights into Histoplasmosis in India: A Tertiary Care Centre Experience

**DOI:** 10.1093/ofid/ofad500.884

**Published:** 2023-11-27

**Authors:** J I N A L SONI, Anmol Jindal, Anivita Aggarwal, Tanvi Batra, A T U L GOGIA

**Affiliations:** Sir Ganga Ram Hospital, New Delhi- 110060, India, Delhi, Delhi, India; Sir Ganga Ram Hospital, New Delhi- 110060, India, Delhi, Delhi, India; Sir Ganga Ram Hospital, New Delhi- 110060, India, Delhi, Delhi, India; Sir Ganga Ram Hospital, New Delhi- 110060, India, Delhi, Delhi, India; Sir Ganga Ram Hospital, New Delhi, India, NEW DELHI, Delhi, India

## Abstract

**Background:**

Serious fungal infections have a significant global impact, affecting millions of lives. Histoplasmosis, commonly reported from the Gangetic Plains of India, presents as a spectrum of varied clinical courses ranging from an acute pulmonary illness to a progressive disseminated histoplasmosis. It is probably underreported due to low index of suspicion, expensive and invasive tests, especially in resource-limited settings like India. In a high tuberculosis burden country, its clinico- radiological resemblance often makes the diagnosis and treatment further challenging for clinicians.This study was conducted to provide deeper insights into the varied clinical presentation and outcomes of Histoplasmosis from a tertiary centre in India.

**Methods:**

We conducted a retrospective observational study including all adult patients who were diagnosed with Histoplasmosis between July 2019 and March 2023 admitted in the Department of Internal Medicine at Sir Ganga Ram Hospital, New Delhi. Their details were collected using electronic records and analysed. They were followed up till the completion of treatment or recent follow up.

**Results:**

We found 8 patients with confirmed histoplasmosis. Majority of the cases were males (87.5%), farmers (37.5%) and belonged to Uttar Pradesh (37.5%),Table 1, Figure 1. Only one patient did not have any attributable underlying risk factor. Fever (100%) and weight loss (50%) were the most common symptoms, Table 2. Anemia, leucopenia and splenomegaly were among the common abnormalities detected, Table 3. Organ-specific involvements were identified by histopathological examination on specific biopsy, Figure 2. Most of the patients (75%) received an initial intravenous course of Liposomal Amphotericin B for 2-3 weeks followed by oral Itraconazole therapy. There were two in-hospital mortalities, out of which one patient expired before the initiation of treatment. Rest of the patients (75%) had clinical recovery on follow up.

**Figure:**
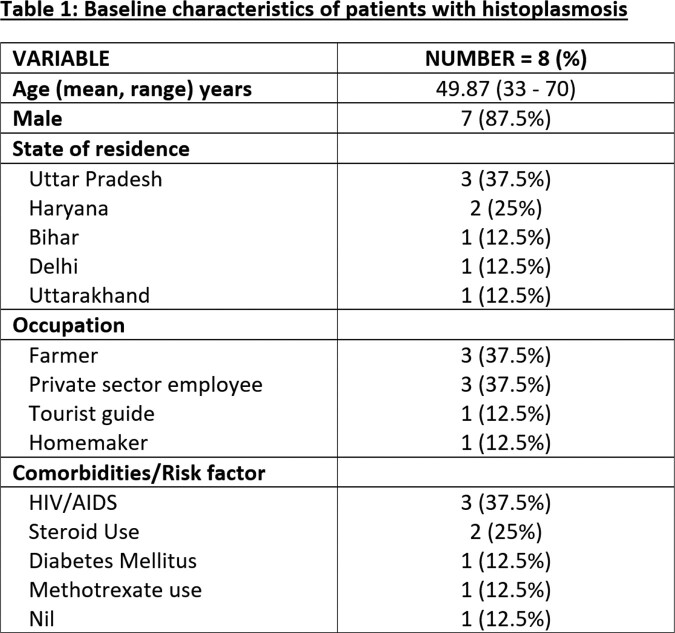

Abbreviations: HIV, human immunodeficiency virus; AIDS, acquired immunodeficiency syndrome

**Figure:**
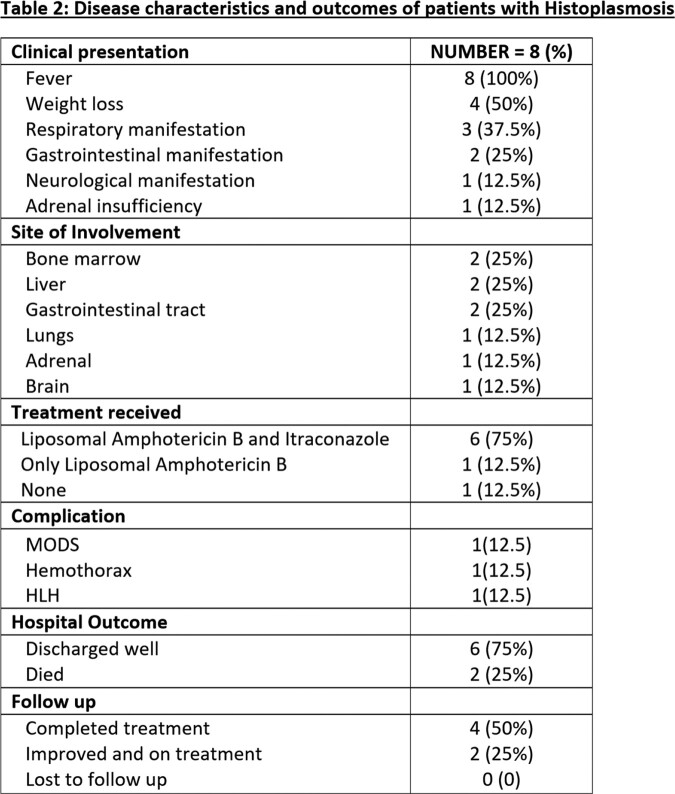

Abbreviations: MODS, multiorgan dysfunction syndrome; HLH, hemophagocytic histiocytosis

**Figure:**
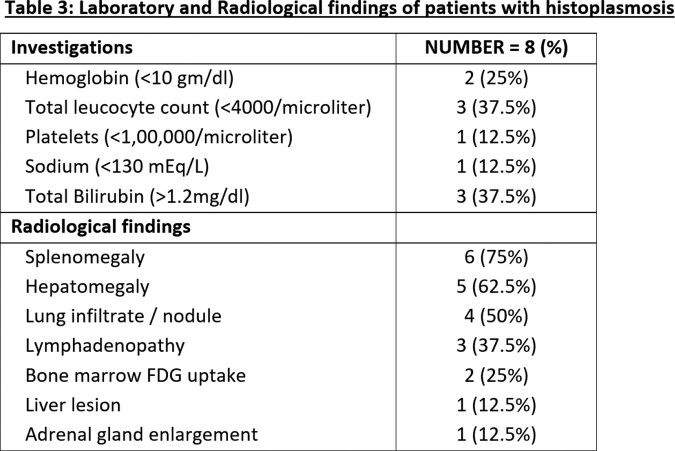

Abbreviations: FDG, fluorodeoxyglucose

**Conclusion:**

Majority of the cases depicted in our study had peculiar diagnostic dilemmas and required a strong clinical suspicion with subsequent invasive procedures to establish the diagnosis. Our study adds to the paucity of data on histoplasmosis and improves the understanding of the disease course among the Indian population.

Distribution of 8 cases of histoplasmosis reported from different states of India

**Figure:**
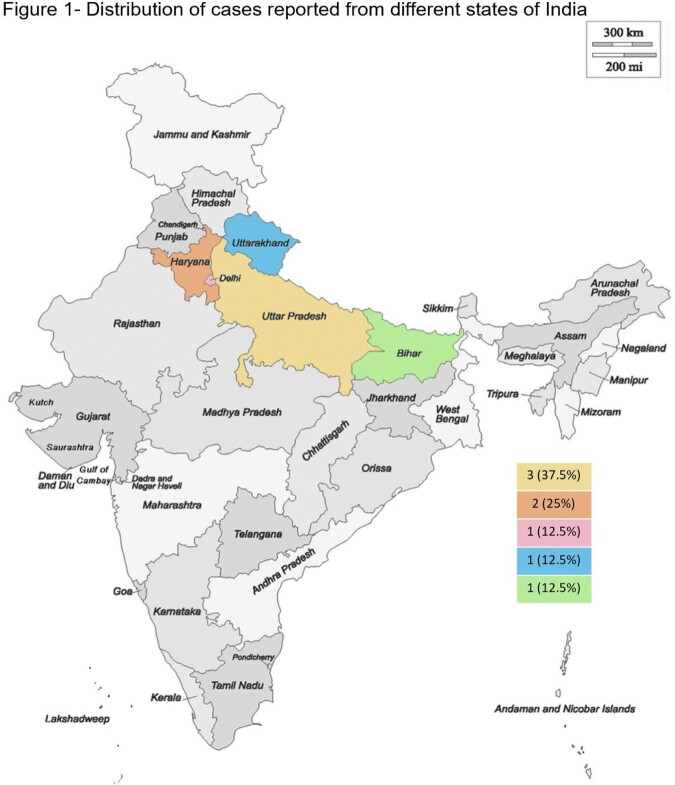

**Figure:**
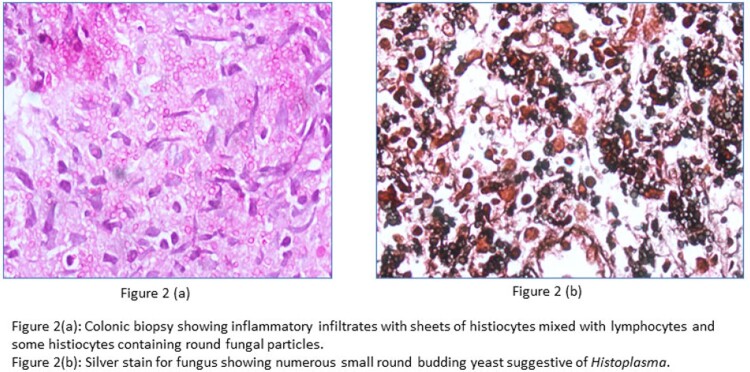

**Disclosures:**

**All Authors**: No reported disclosures

